# Oritavancin as sequential therapy for Gram-positive bloodstream infections

**DOI:** 10.1186/s12879-023-08725-8

**Published:** 2024-01-24

**Authors:** Williams Monier Texidor, Matthew A. Miller, Kyle C. Molina, Martin Krsak, Barbara Calvert, Caitlin Hart, Marie Storer, Douglas N. Fish

**Affiliations:** 1 Department of Clinical Pharmacy, University of Colorado Skaggs School of Pharmacy and Pharmaceutical Science, Aurora, CO USA; 2 Department of Pharmacy-Infectious Diseases, University of Colorado Hospital, Aurora, CO USA; 3 Children’s Hospital Colorado, Aurora, CO USA; 4 Department of Medicine, Division of Infectious Diseases, University of Colorado School of Medicine, Aurora, CO USA

**Keywords:** Oritavancin, Bloodstream Infection, Gram-positive, Infective endocarditis, Sequential therapy

## Abstract

**Background:**

Oritavancin, a long-acting lipoglycopeptide approved for use in acute bacterial skin and skin structure infections, has limited data evaluating use in serious infections due to Gram-positive organisms. We aimed to assess the effectiveness and safety of oritavancin for consolidative treatment of Gram-positive bloodstream infections (BSI), including infective endocarditis (IE).

**Methods:**

We conducted a retrospective cohort study evaluating adult patients admitted to University of Colorado Hospital from March 2016 to January 2022 who received ≥ 1 oritavancin dose for treatment of Gram-positive BSI. Patients were excluded if the index culture was drawn at an outside facility or were > 89 years of age. The primary outcome was a 90-day composite failure (clinical or microbiological failure) in those with 90-day follow-up. Secondary outcomes included individual components of the primary outcome, acute kidney injury (AKI), infusion-related reactions (IRR), and institutional cost avoidance.

**Results:**

Overall, 72 patients were included. Mean ± SD age was 54 ± 16 years, 61% were male, and 10% had IE. Organisms most commonly causing BSI were *Staphylococcus aureus* (68%, 17% methicillin-resistant), followed by *Streptococcus* spp. (26%), and *Enterococcus* spp. (10%). Patients received standard-of-care antibiotics before oritavancin for a median (IQR) of 11 (5–17) days. Composite failure in the clinically evaluable population (n = 64) at 90-days occurred in 14% and was composed of clinical and microbiological failure, which occurred in 14% and 5% of patients, respectively. Three patients (4%) experienced AKI after oritavancin, and two (3%) experienced an IRR. Oritavancin utilization resulted in earlier discharge for 94% of patients corresponding to an institutional cost-avoidance of $3,055,804 (mean $44,938/patient) from 1,102 hospital days saved (mean 16 days/patient).

**Conclusions:**

The use of oritavancin may be an effective sequential therapy for Gram-positive BSI to facilitate early discharge resulting in institutional cost avoidance.

**Supplementary Information:**

The online version contains supplementary material available at 10.1186/s12879-023-08725-8.

## Introduction

Bloodstream infections (BSI), including infective endocarditis (IE), due to Gram-positive organisms often require prolonged intravenous (IV) antimicrobial therapy resulting in considerable hospital length of stay (LOS) and healthcare costs [1, 2]. Several treatment modalities for patients requiring prolonged courses exist, including continued inpatient stay for IV antibiotics, outpatient parenteral antimicrobial therapy (OPAT), or discharge with oral antibiotics [3]. However, not all patients are eligible for these approaches owing to psychosocial factors or housing instability [2, 4]. Continued hospitalization and OPAT both confer significant risks of complications (e.g., thrombophlebitis, infection, line dysfunction) and require substantial healthcare resources (e.g., lab monitoring, care coordination, and personnel) [5–8].

Oritavancin is a long-acting lipoglycopeptide antimicrobial with in vitro activity against a variety of Gram-positive organisms, including *Staphylococcus aureus* (methicillin-susceptible [MSSA] and methicillin-resistant [MRSA]), *Enterococcus* spp. (including vancomycin-resistant enterococci [VRE] due to VanA), and *Streptococcus* spp. Oritavancin has received FDA approval to treat acute bacterial skin and skin structure infections (ABSSSI) due to MSSA, MRSA, beta-hemolytic *Streptococci*, *Streptococcus anginosus* group, and vancomycin-susceptible *Enterococcus faecalis* [9]. Given a prolonged half-life of ~ 245 h, oritavancin maintains a free plasma concentration above the minimum inhibitory concentration for many Gram-positive organisms for several weeks [10]. These characteristics position oritavancin as an attractive option to extend the treatment of Gram-positive BSI beyond discharge. Preliminary studies evaluating oritavancin for the treatment of complicated Gram-positive infections appear promising; however, data for the treatment of BSI remain limited [11–14]. Therefore, this study aimed to assess the effectiveness and safety of oritavancin as sequential therapy for BSI, including IE, due to Gram-positive organisms.

## Methods

We conducted a retrospective, observational cohort study evaluating adult patients at the University of Colorado Hospital from March 2016 to January 2022. Included subjects received at least one dose of oritavancin for the treatment of a BSI due to any Gram-positive organism. Patients were excluded if they were > 89 years of age as required by the local Institutional Review Board (IRB), received oritavancin or had an index blood culture drawn at an outside hospital. This study received IRB approval before study initiation.

The electronic health record (EHR; Epic, Verona, WI) was queried for oritavancin administrations within the study period. The oritavancin product in use at the institution was the original formulation (Orbactiv). Data extracted from the EHR included patient demographics, comorbidities, infection and treatment information, length of stay, adverse events, and outcomes, including hospital readmission, re-infection, and mortality. The index organism(s) was the Gram-positive organism(s) referred to in the EHR by a treating physician as the etiology of the BSI. Infections in which more than one pathogenic organism was identified (including Gram-negatives and anaerobes) were classified as polymicrobial. Common commensal organisms such as coagulase-negative *Staphylococcus* spp. were considered contaminants and excluded if cultured in only one of two blood culture sets. *S. aureus* BSI was defined as complicated by one of more of the following: lack of defervescence by 72 h after initiating antibiotic therapy, metastatic sites of infection, repeat positive blood cultures with the same organism after 48 h of therapy, presence of implanted prosthesis/devices, previous *S. aureus* BSI within 90-days, prior IE, active immunosuppression including neutropenia or a prior organ transplant, or catheter-related BSI without catheter removal within the first 72 h after positive blood cultures [15, 16]. IE was categorized as definitive or possible according to the modified Duke criteria [17]. Acute kidney injury (AKI) was defined as meeting at least stage I injury according to the 2012 Kidney Disease: Improving Global Outcomes Clinical Practice Guideline for Acute Kidney Injury [18].

### Outcomes

The primary endpoint was a 90-day composite failure, comprised of clinical or microbiological failure within 90 days from index culture in the clinically evaluable (CE) patient population. The clinically evaluable patient population includes those with follow-up within the healthcare system at 90 days. Clinical failure was defined as the initiation of a Gram-positive antibiotic after oritavancin administration, infection-related readmission due to the index infection, and all-cause mortality. Microbiological failure was defined as identifying a new BSI with the same species as the index organism. Secondary endpoints were the individual components of the primary endpoint, incidence of AKI, and incidence of infusion-related reactions. Adjudication of effectiveness outcomes was performed by an ID physician (M.K.).

### Cost analysis

In patients discharged early (i.e., documented antibiotic end-date was after their discharge date), the number of hospital days saved associated with oritavancin use was calculated by subtracting the date of discharge from the documented antibiotic end date. The cost avoidance of reduced hospital days per patient was calculated by multiplying the hospital days avoided by the average cost of an inpatient stay in Colorado ($3,047/day) [19]. Total institutional cost avoidance was determined by subtracting the average wholesale price (AWP) of oritavancin for each dose administered from the cost avoidance achieved from reduced hospital days. The AWP used for oritavancin at the time of the study was $2,626 and $3,939 for 800 mg and 1,200 mg doses, respectively [20].

## Results

Overall, 72 patients were included in the study. Reasons for exclusion were receipt of oritavancin (n = 11) or index blood cultures (n = 7) at an outside hospital, age < 18 or > 89 years (n = 1), or not completing the oritavancin infusion (n = 1). Baseline characteristics are displayed in Table 1. The mean ± standard deviation (SD) age was 54 ± 16 years, and 61% (n = 44/72) of patients were male. The median (IQR) Charlson Comorbidity Index was 3 (1–5), and 17% (n = 12/72) of patients were admitted to ICU at the time the index culture was collected. *S. aureus* was the causative organism in 68% (n = 49/72) of cases, 51% (n = 25/49) of which were complicated, and 24% (n = 12/49) were MRSA. Other causative organisms included *Streptococcus* spp. (26%, n = 19/72), *Enterococcus* spp. (10%, n = 7/72), and coagulase-negative *Staphylococcus* spp. (8%, n = 6/72); 28% (n = 20/72) had a polymicrobial BSI. Seven patients (10%) had IE, of which five were classified as definitive IE, and two involved a prosthetic valve. All patients received standard-of-care antibiotics before oritavancin, with a median (IQR) duration of 11 (5–17) days. Among those with follow-up blood cultures, all but one patient received oritavancin after clearance of blood cultures. The single patient without culture clearance prior to oritavancin was being treated for refractory VRE BSI, where oritavancin was instituted as salvage therapy.

**Table 1:** Patient demographics and treatment details

**Variable**	**N = 72**
Age (years), mean (SD)	54 (16)
Male sex, n (%)	44 (61)
Race, n (%)	
White	46 (64)
Hispanic	15 (21)
Black	5 (7)
Body mass index (kg/m^2^), mean (SD)	28 (8)
Charlson Comorbidity Index, median (IQR)	3 (1–5)
Index organism(s), n (%)	
*S. aureus*	49 (68)
Methicillin-resistant	12 (17)
*Streptococcus* spp.	19 (26)
Beta-hemolytic	13 (18)
Viridans group & other *Streptococcus* spp.^a^	6 (8)
*Enterococcus* spp.	7 (10)
Vancomycin-resistant	4 (6)
Coagulase-negative *Staphylococcus* spp.	6 (8)
Other^b^	4 (6)
Infectious diseases consult, n (%)	71 (99)
Prior antibiotics therapy, n (%)	
Vancomycin	64 (89)
Ceftriaxone	36 (50)
Cefazolin	35 (49)
Linezolid	18 (25)
Ampicillin	10 (14)
Ceftaroline	7 (10)
Daptomycin	6 (8)
Days of antibiotics prior to oritavancin dose, median (IQR)	11 (5–17)
Bloodstream infection clearance prior to oritavancin administration ^c^, n (%)	69 (99)
Oritavancin dose, n (%)	
800 mg once	4 (6)
1200 mg once	53 (74)
1200 mg, followed by 1200 mg	5 (7)
1200 mg, followed by 800 mg	10 (14)
Hospital LOS, median (IQR)	17 (8–24)

^a^Viridans group streptococcus includes S. anginosus group (1), S. salivarius group (2), *S. pneumoniae* (1), *S. mitis/oralis* (1), *S. mutans* (1)

^b^Other organisms include *Aerococcus viridans* (1), *Corynebacterium amycolatum* (1), *Cutibacterium acnes* (1), *Granulicatella adiacens* (1)

^c^Only calculated for patients with follow-up blood cultures (n = 70)

In total, 8 patients had loss to follow-up, leaving 64 patients in our CE population. Composite failure at 90-days in the CE population was 14% (n = 9/64, Table 2). Clinical and microbiological failures occurred in 14% (n = 9/64) and 5% (n = 3/64), respectively. Two patients with microbiological failure died within 90 days, and one was started on a Gram-positive agent for presumed treatment failure. All-cause mortality occurred in 13% of patients. Ninety-day infection-related readmission was observed in 11% of patients, only one of which was due to recurrence with the index organism. Three patients experienced AKI after oritavancin occurring between 4 and 23 days after oritavancin administration. Two of the three patients met KDIGO stage 3 criteria. Only two patients had an infusion-related reaction. Complete case descriptions of patients who met the composite failure definition are presented in Additional file 1: **Table ****s1**.

**Table 2:** Effectiveness in clinically evaluable patients and secondary outcomes at 90-days

**Primary Outcome**	
Composite failure at 90-days^a^, n (%)	9 (14)
**Secondary Outcomes**	
Clinical failure^a^, n (%)	9 (14)
All-cause mortality	8 (13)
Initiation of Gram-positive antibiotic for presumed failure	1 (2)
Index Infection-related readmission	1 (2)
Microbiological failure^a ,^ n (%)	3 (5)
Infection-related readmission^b^, n (%)	8 (11)
Acute Kidney Injury^b^, n (%)	19 (26)
Prior to Oritavancin administration	19 (26)
AKI after Oritavancin administration	3 (4)
Infusion-related reactions^b^, n (%)	2 (3)
Loss to follow-up^b^, n (%)	8 (11)

^a^Among 64 patients in clinically evaluable cohort (had follow-up through 90 days)

^b^Among 72 patients in total cohort

Oritavancin utilization resulted in earlier discharge for 94% of patients in the overall cohort (n = 68/72). Eighty-one doses of oritavancin led to 1,102 hospital days saved (mean 16 days/patient), corresponding to an estimated total institutional cost-avoidance of $3,055,804 over the 6-year study period (mean $44,938/patient).

## Discussion

To our knowledge, this is the largest retrospective cohort evaluating the use of oritavancin solely in BSI, including complicated BSI. This study of patients undergoing treatment for Gram-positive BSI with oritavancin demonstrated favorable rates of clinical and microbiological cure. Overall, clinical failure in the CE population was low (14%) and in line with prior studies evaluating conventionally used therapies, vancomycin or daptomycin [21, 22]. Likewise, microbiological failure and infection-related readmission were uncommon, with only one patient experiencing infection-related readmission due to the index infection. Oritavancin use allowed for earlier discharge in most patients resulting in significant cost-avoidance while adverse drug events following oritavancin administration were infrequent. Overall, our findings suggest that oritavancin may be a reasonable alternative to standard therapies when used as sequential therapy after blood culture clearance.

Although limited, real-world use of oritavancin for complex infections has generally demonstrated promising effectiveness outcomes with a favorable safety profile [11–14]. Schulz and colleagues previously demonstrated success or improvement in all 17 patients treated with oritavancin for documented or presumed osteomyelitis or intravascular infections caused by Gram-positive organisms [14]. Another series of 10 patients with invasive Gram-positive infections demonstrated 70% treatment success with oritavancin after initial standard-of-care antimicrobials [13]. Nonetheless, limited data exist describing oritavancin utilization for the primary management of BSI. None of the patients in the registrational trials had BSI. Although the real-world CHROME registry evaluated 446 patients with ABSSSI and other Gram-positive infections, only seven patients had BSI, and their outcomes were not directly reported [11, 23, 24].

Despite limited data with oritavancin in BSI, other long-acting lipoglycopeptides have also shown promising early findings. A retrospective study evaluating dalbavancin in BSI and IE suggested favorable outcomes [25]. An analysis of sequential dalbavancin compared to standard-of-care therapy at our institution suggested similar effectiveness between the two approaches [26]. Further, the use of dalbavancin was associated with reduced central catheter utilization, and shorter length of stay. Although a direct comparison to that study is not feasible, the overall low rate of overt clinical failure in both studies adds to the existing literature supporting the expanded role of long-acting lipoglycopeptide antimicrobials in treating invasive infection following clearance of BSI [25, 26]. Results from Dalbavancin as an Option in the Treatment of Staphylococcus Aureus Bacteremia (DOTS) trial are anxiously anticipated to further define the role of long-acting lipoglycopeptides in this setting (ClinicalTrials.gov identifier: NCT04775953).

Similar to the total cost-saving reported in this cohort (average $44,938/patient), multiple studies have shown the financial benefit of oritavancin use by reducing hospital length of stay or admission avoidance [5, 6, 27]. A study by Brownell and colleagues evaluated 75 patients with ABSSSI treated with oritavancin and reported a per-patient average cost avoidance of $4,708. Similarly, a cost-minimization model comparing inpatient vancomycin to outpatient oritavancin for treatment of uncomplicated ABSSSI estimated cost savings between $1,752 to $6,475 per patient, depending on the number of patient comorbidities. In that analysis, budget neutrality was maintained with modeled readmission rates of up to 38%, demonstrating the insensitivity of cost avoidance with respect to readmission [6]. Alike this study, we have previously shown that dalbavancin used as sequential therapy results in reduced hospital length of stay, corresponding to an average cost avoidance of $17,204 per patient [28]. The difference in cost avoidance between the current study and those mentioned prior may be attributed to a larger proportion of diseases requiring longer treatment courses. Additionally, the median (IQR) days on antibiotics before oritavancin was shorter in the current study [11 (5–17) vs. 13 (7-24.5)], possibly influenced by earlier implementation of long-acting lipoglycopeptide as sequential therapy at our institution. Despite recent promising data with long-acting agents for treating severe Gram-positive infections, formal pharmacoeconomic comparisons have yet to be performed. Additional studies are needed to determine the optimal long-acting lipoglycopeptide, timing of therapy, and whether combination antimicrobial therapy for *S. aureus* and *Enterococcus spp*. can expedite BSI clearance and provide a lower incidence of complication and earlier readiness for patient discharge.

This study should be interpreted with consideration of several limitations. The retrospective, non-comparative, single-center design may limit the generalizability of this data. The treatment of patients with oritavancin reflects our institutional practice and may select for patients with relatively uncomplicated BSI. Although most patients were treated for MSSA infection, all were pre-treated with standard-of-care antimicrobials, and oritavancin was reserved for consolidation therapy after blood culture clearance. Given the population described in this cohort, administration of one to two doses of oritavancin can ensure the completion of therapy in patients who may not follow up with care after discharge.

This study suggests an expanded role of oritavancin as consolidation therapy for Gram-positive BSI in select patients. In addition, oritavancin appears to have a favorable safety profile and can result in significant institutional cost avoidance.

### Electronic supplementary material

Below is the link to the electronic supplementary material.

Supplementary Material 1

## Data Availability

The researchers confirm the accuracy of the data provided for the study and its available from the corresponding author on reasonable request.
