# Supplementary material for: Oritavancin as sequential therapy for Gram-positive bloodstream infections
Source: BMC Infect Dis. 2024 Jan 24;24:127. doi: 10.1186/s12879-023-08725-8 (PMC10807122; doi:10.1186/s12879-023-08725-8)
Supplement: Supplementary file 1 — Supplementary Material 1 [file 12879_2023_8725_MOESM1_ESM.docx]

**Table s1: Description of Patients with Composite Failure**

| **Age/Sex** | **Micro-organism/Infection** | **Composite Failure** | **Adverse event** | **SOC abx duration in weeks before** **ORI/ORI** **dose in mg (# of doses)** | **Comments** |
| --- | --- | --- | --- | --- | --- |
| 58/F | MSSA/bacteremia | Clinical and microbiological failure | None | 5/1200 (1) | The original admission MRI did not indicate infection. Patient received 1 dose of ORI and was then discharged. Patient readmitted due to complaint of back pain. Repeat MRI showed OM and epidural phlegmon, so cefazolin was started. |
| 81/F | VRE/bacteremia | Clinical failure | None | 1/1200 (2) | Refractory MDS that progressed to AML and received 2 cycles of LDAC/cladribine/venetoclax. Primary cause of death was documented as septic shock. Secondary cause of death was pneumonia and endocarditis. Oritavancin used for VRE coverage given presence in urine and lungs. |
| 55/M | MSSA/endocarditis | Clinical failure | AKI prior to ORI dose | 1.5/1200(1) | Endocarditis and immunologic phenomenon (roth spots and Janeway lesions). Patient discharged on ORI. Readmitted a day later due to large left temporal-parietal-occipital IPH requiring 24-hour dependent care. Family decided on palliative extubation. Primary cause of death determined to be cardiac arrest post-extubation. |
| 20/M | VSE/endocarditis | Clinical failure | AKI prior to ORI dose | 5/1200(1) | One dose of ORI was presumed to cover the patient through the end of a 6-week course. Patient readmitted 6 weeks after ORI dose and was found to have septic PE with cavitation on CT A/P. Blood cultures grew MSSA 4/4, and ceftriaxone was started. Vancomycin, ampicillin and gentamicin were added later on. Primary cause of death was documented as IVDU leading to recurrent endocarditis and cardiogenic shock. Restart of Gram-positive agents was beyond the presumed duration of ORI coverage and with non-index pathogen; therefore, patient was not counted towards that outcome. |
| 60/M | VSE/bacteremia | Clinical and microbiological failure | AKI prior to ORI dose | 3/1200(1) | AML patient with recurrent VSE bacteremia, post-transplant D+32 with no sign of engraftment went into refractory septic shock after moving to comfort care. That same day, patient was initiated on ORI and died soon after. |
| 30/F | *S. pseudintermedius*/ bacteremia | Clinical failure | None | 0.3/1200(1) | Patient with alcoholic cirrhosis grew *E. coli* in blood cultures from SBP leading to septic shock. Documented cause of death was GNR septic shock from decompensated cirrhosis and alcohol use disorder. |
| 54/M | MRSE and VSE/bacteremia | Clinical and microbiological failure | AKI stage 3 prior to ORI dose that continued after the dose | 5/1200(1) then 800(1) | History of liver/kidney transplant 4 years prior, autologous HCT followed by allogeneic HCT during the previous year. Presented with VRE CLABSI breakthrough while on daptomycin with associated DVT. Two doses of ORI given 4-days apart. VRE was persistently detected by blood cultures after ORI given. Etiology of death on January 14^th^ is listed as septic shock due to MRSE/VSE bacteremia. |
| 21/F | MRSA/bacteremia | Clinical failure | None | 2/1200(1) | Lupus nephritis patient with history of multiple PEA arrests due to hyperkalemia. Received ORI for MRSA bacteremia. Readmitted and found to have *E. coli* bacteremia. Documented cause of death is septic shock due to GNR bacteremia. |
| 79/F | MSSA/bacteremia | Clinical failure | None | 0.5/1200(1) | Lung cancer patient with malignant pleural effusion being treated for MSSA bacteremia with two planned doses of ORI. Before receiving the 2nd dose, the patient was readmitted for non-infectious causes and decided to switch to cefazolin while hospitalized. One month later the patient's daughter stated the patient had died. The cause of death was not described in the EHR. |
| 79/M | Beta hemolytic streptococci/bacteremia | Loss to follow up | None | 0.7/800(1) | Patient with bioprosthetic valve replacement was treated for group B streptococcal bacteremia. Upon negative TEE findings, a one-time inpatient dose of ORI was chosen over OPAT to cover for a total of 2 weeks. Post index admission, the patient did not have follow-up appointments within our health system but was discharged with instructions to follow up at another health system for initiation of HD. |
| 36/M | MSSA/bacteremia | Loss to follow up | None | 0.6/1200(2) | Methamphetamine use disorder, mood disorder, and homelessness admitted for elbow pain and found to have abscess and MSSA bacteremia treated with ORI. Patient did not have follow up post index admission. |
| 60/M | *Aerococcus viridans*/endocarditis | Loss to follow up | None | 4/1200(1) | Patient with alcohol use disorder admitted after falling from bed resulting in facial trauma and found to have polymicrobial bacteremia with associated endocarditis. Patient was not an OPAT candidate and therefore received ORI and did not have follow-up post index admission. |
| 55/F | MSSA/bacteremia | Loss to follow up | None | 4/1200(1) then 800(1) | Patient with rheumatoid arthritis on methotrexate developed MSSA/Stenotrophomonas septic arthritis and MSSA bacteremia. Source control was unable to be performed, so plan was to treat with IV antibiotics and two doses of ORI with estimated coverage of 2 months. Patient did not have insurance and therefore did not have follow-up post index admission. |
| 54/F | MSSA and beta-hemolytic streptococci/bacteremia | Loss to follow up | None | 0.7/1200(1) | Patient with rheumatoid arthritis and systemic lupus erythematous with intra-abdominal abscess s/p colectomy/colostomy and MSSA and beta hemolytic streptococcal bacteremia with plans to treat for 2 weeks. Patient desired to leave hospital AMA; therefore one dose of ORI was given to complete the remaining course of therapy. Patient did not have follow up post index admission |
| 43/F | *Streptococcus salivarius group*/bacteremia | Loss to follow up | None | 2/1200(1) | Decompensated cirrhosis secondary to untreated HCV and *Staphylococcus* endocarditis a year prior was found to have *S. salivarius* bacteremia treated with 2 weeks of IV and one dose of ORI to for a total of 4 weeks of therapy. Patient did not have adequate shelter and therefore remained hospitalized 2 weeks post ORI dose at which time she was afebrile with stable labs and hemodynamic. |
| 65/M | MRSA and *Strep dysgalactiae* /bacteremia | Loss to follow up | AKI prior to ORI dose | 2/1200(1) | Patient has history of alcohol use disorder and cholesteatoma s/p tympan mastoidectomy leading to bacteremia from chronic otitis and mastoiditis with associated maggot infection. The decision was made to complete 4 weeks course with one dose of ORI after 2 weeks of vancomycin given adherence issues with oral agents and not an OPAT candidate. Patient was discharged two days later and had no follow-up after index admission. |
| 38/F | MSSA/bacteremia | Loss to follow up | None | 4/800(1) | Homeless and substance use disorder with MSSA bacteremia likely from sternal fracture treated with cefazolin. Patient received one dose of ORI prior to discharge to complete a 6-week course of antibiotics and did not have follow-up after index admission |

Abbreviations: Abx: antibiotic, AKI: acute kidney injury, CLABSI: catheter-line associated bloodstream infection, CT A/P: CT abdomen and pelvis, DVT: deep vein thrombosis, EHR: electronic health record, GNR: Gram-negative rod, HCT: hematopoietic stem-cell transplant, HD: hemodialysis, IPH: intraparenchymal hemorrhage, LDAC: low-dose cytarabine, MSSA: methicillin-susceptible *S. aureus,* MDS: myelodysplastic syndrome, OM: osteomyelitis, OPAT: outpatient parenteral antimicrobial therapy, ORI: oritavancin , PE: pulmonary emboli, PEA: pulseless electrical activity, SBP: spontaneous bacterial peritonitis, SOC: standard of care, S/P: status post
